# Mobile App for Improving the Mental Health of Youth in Out-of-Home Care: Development Study Using an Intervention Mapping Approach

**DOI:** 10.2196/64681

**Published:** 2024-11-21

**Authors:** Jinyoung Park, Jungeun Lee, Dabok Noh

**Affiliations:** 1 College of Nursing Yonsei University Seoul Republic of Korea; 2 College of Social Science Dankook University Gyeonggi-do Republic of Korea; 3 College of Nursing Eulji University Gyeonggi-do Republic of Korea

**Keywords:** out-of-home youth, mental health intervention, mobile app, intervention mapping, youth, mental health, mHealth, mobile health, app, interview, need, focus group, emotion, emotional, young adult, independent living, emotional support, tool, emotion regulation, user, app usage

## Abstract

**Background:**

Youth in out-of-home care encounter substantial mental health challenges because of the absence of stable family and social support systems. Their vulnerability is heightened by trauma, neglect, and abuse. They struggle, especially when transitioning to independent living, coping with loneliness, anxiety, and pressure.

**Objective:**

This study aimed to develop a mobile app with high accessibility and long-term continuous effects to support independent living and improve mental health among youth in out-of-home care. The approach used was the systematic and step-by-step intervention mapping (IM) framework.

**Methods:**

The program was created using the IM framework and had 6 steps. Drawing from data from individual and focus group interviews and literature reviews, we developed a logical model of the problem. We established program outcomes and objectives, defining performance objectives and variable determinants. We identified theoretical and evidence-based methods that influence determinants. The app design integrated these methods into practical applications, allowing for the creation of self-management and emotional support tools. The development process included ongoing discussions between app designers and the research team to ensure that user needs and preferences were addressed.

**Results:**

Individual interviews and focus group discussions revealed challenges in managing daily routines and regulating emotions. The program design was based on the transtheoretical model, social cognitive theory, and elaboration likelihood model. Key features included goal setting, structured routines, emotion recognition flashcards, character models demonstrating emotion regulation strategies, verbal persuasion, and self-monitoring tools to support habit formation and emotion regulation. An implementation plan was developed to facilitate the app’s adoption, execution, and maintenance, while an evaluation plan was established, including app usage analytics, user logs, and feedback surveys. A randomized controlled trial will be conducted to assess the app’s impact on mental health outcomes, focusing on reducing anxiety and depressive symptoms, improving emotion regulation, and enhancing daily living skills.

**Conclusions:**

The IM framework was beneficial in developing a mobile app to enhance the mental health of youth in out-of-home care. The study produced a program grounded in theory and evidence that caters to the needs of these individuals. Further research should aim to verify the app’s effectiveness in real-world settings and refine it continuously based on user input.

## Introduction

In South Korea, “out-of-home youth” refers to individuals aged 9-24 years who have been separated from their guardians due to family conflicts, abuse, violence, neglect, family dissolution, or elopement and thus require social protection and support [1].

Youth in out-of-home care settings, such as child welfare facilities, orphanages, group homes, and youth shelters, are vulnerable to mental health issues due to the absence of stable family and social support systems [2,3]. This vulnerability is worsened by trauma, neglect, and abuse, which are prevalent among these youth [4-6]. As a result, they face long-term mental health challenges, and recent evidence reports that individuals who have lived in out-of-home care experience higher mortality rates in adulthood, largely due to increased rates of self-harm, accidents, and other mental health and behavioral factors [7].

These youth are especially vulnerable when transitioning from out-of-home care to living independently [4,5]. They often fail to make realistic and specific plans for independence, resulting in anxiety and pressure [8]. When individuals leave shelters, they face a sudden lack of support, leading to economic challenges such as housing instability, inadequate job opportunities, and financial hardship [9,10]. During the transition from youth to adulthood, individuals often face various crises, and the lack of a social support system can intensify feelings of isolation, loneliness, and despair, ultimately impacting their mental health [4,5,8]. Similarly, unaccompanied youth who are homeless experience high rates of obesity and poor diet quality, with many having deficiencies in essential nutrients. This exacerbates their physical and mental vulnerabilities, contributing to long-term health issues [11].

Various interventions have been proposed to address the mental health challenges of out-of-home care youth [12]. Some studies have attempted interventions for mental health, such as art therapy [13], cognitive behavior therapy [14,15], and family therapy [16]. These interventions predominantly rely on face-to-face counseling. Furthermore, a systematic review of interventions for care-experienced children and young people found that over half of the reviewed studies were conducted in the United States, raising concerns about regional bias and the generalizability of these interventions to other contexts [17].

In addition, according to a meta-analysis by Trubey et al [12], interventions aimed at improving the mental health of children and young people in out-of-home care showed significant positive effects on depression, anxiety, and social functioning difficulties in the short term (0-6 months); however, there was no evidence of effectiveness for long-term outcomes (>6 months) [12]. Furthermore, youth in out-of-home care often lose contact with the facilities when they become independent, leading to inconsistent management and heightened vulnerability [4,5]. Hence, youth living independently should receive ongoing and structured assistance.

The intervention mapping (IM) framework is effective in designing systematic, evidence-based health interventions [18]. For example, Svendsen et al [19] developed a self-management app for low back pain, improving adherence and outcomes through tailored advice. Hadjiconstantinou et al [20] addressed psychological and behavioral factors in their type 2 diabetes management program, and Wong et al [21] enhanced recovery and autonomy with a stroke self-management intervention. These studies demonstrate the use of IM in designing various health interventions.

In this study, we created a mobile app to enhance independent living, accessibility, and long-term mental health benefits for youth in out-of-home care based on the IM approach.

## Methods

### Overview

A mobile app was developed from October 2023 to June 2024 to enhance the mental health of youth in out-of-home care. The app was created using the IM approach, which is a systematic method that integrates theoretical and empirical evidence to create impactful health promotion programs tailored to the needs of youth in out-of-home care [18]. It involves engaging stakeholders throughout the program development process to ensure that their perspectives and needs are considered [22]. It consists of six steps: (1) a logical model of the problem, (2) program outcomes and objectives (logical model of change), (3) program design, (4) program production, (5) program implementation plan, and (6) evaluation [18].

### Step 1: Logical Model of the Problem

#### Establish and Work With a Planning Group

Initially, we established a multidisciplinary planning group to incorporate expertise from various domains, with a particular focus on mental health improvement, youth care, and social welfare. The group consisted of a psychiatric and mental health nurse with a PhD, a PhD graduate in child and family studies, 2 social workers serving as directors at youth shelters, and a social worker employed at a youth shelter. The planning group worked closely with an app development team, which included an app developer, a designer, and a lead app developer who also served as the server and web developer, to ensure that the digital platform was appropriately tailored to meet the needs of youth in out-of-home care. To gather feedback directly from the target population for this intervention without exposing them to the pressures or responsibilities of participating in a formal planning group, we decided not to include youth in the planning group. Instead, we conducted individual interviews to assess their needs.

#### Needs Assessment

We conducted a needs assessment to analyze respondents’ mental health and quality of life issues, as well as their underlying causes. The methods used for this assessment included conducting a literature review, holding individual interviews with youth living in youth shelters, and organizing focus group interviews with shelter workers.

The target population for this intervention was youth aged 18-25 years who receive out-of-home care at youth shelters and are preparing for independent living after leaving such care. This focus was motivated by two main reasons. First, previous research indicated that youth in out-of-home care are vulnerable to mental and physical health issues due to the absence of stable family and social support systems [2,3,7]. Second, youth in out-of-home care in South Korea must transition to independent living between the ages of 18 and 25 years. Thus, our target population consisted of individuals aged 18 to 25 years who are in the process of transitioning to independence.

#### Individual Interviews With Youth

We conducted individual interviews with 5 youths residing in 2 youth shelters. The participants were chosen purposefully to include those who could share their experiences living away from family and preparing for independence. The interview questions were designed to gather insights into the participants’ experiences with mental health difficulties and their specific needs, which were crucial in structuring the intervention. The participants were asked questions such as “Have you ever experienced psychological or emotional difficulties?” and “Have you sought help during tough times?” In addition to these, they were asked about concerns related to independent living, including “What is your biggest worry as you prepare to live independently?” and “What challenges do you expect to face in your daily life after leaving the youth shelter?” as well as “What emotional or psychological challenges do you anticipate after becoming independent?” To explore their perspectives on digital interventions, participants were asked, “If there was a mental health program available through a mobile app, what kind of support would you want?” Finally, they were asked, “What advice would you like to give to the program developers?” and “Is there anything else you would like to add?” The interviews, conducted in the counseling rooms of the shelters, lasted around 1 hour.

#### Focus Group Interviews With Shelter Workers

We also conducted 2 focus group interviews with 10 shelter workers from 2 youth shelters. These workers were selected based on their experience of working at youth shelters for 1 year or more and their deep understanding of the experiences of out-of-home youth. The interviews focused on discussing the mental health challenges faced by these youth, instances where assistance was sought, and the need for intervention programs. To begin the interviews, the participants were asked to introduce themselves by sharing how long they have worked in their roles and a brief overview of their responsibilities. We then explored perspectives on the mental health status of youth residing in youth shelters, asking, “What do you think about the mental health condition of these youths?” As the conversation progressed, we delved into specific experiences while working in youth shelters: “Have you observed any cases of mental health difficulties among these youths?” Thereafter, we discussed experiences in assisting youth in need: “Have you encountered any instances where a youth sought help with mental health issues?” To further understand their insights, we asked, “In your opinion, what do you think is beneficial for the mental health of youths?” We also discussed the potential of mobile applications in this context, asking, “If a mental health promotion program were to be delivered through a mobile app, in what ways do you think it could be helpful?” To conclude the interviews, we asked, “What advice would you like to give to the program developers?” and “Is there anything else you would like to add?” Each interview lasted about 1 hour and was conducted in the meeting rooms of the two youth shelters.

All interviews were audio-recorded and subsequently transcribed. The interviews were exploratory, focusing on gathering real-world needs and collaborating with stakeholders. Insights obtained from the open-ended, semistructured interviews were integrated iteratively, and any emergent trends were reviewed with the planning group.

Finally, we outlined the intervention’s context, which includes the population, community, and setting. In the final step of Phase 1, we defined the program’s goals.

### Step 2: Program Outcomes and Objectives: Logical Model of Change

In the second step, we identified the expected outcomes from the results of Step 1. We defined the performance objectives for these outcomes and chose their determinants. A matrix was created to outline the change objectives that would impact the determinants and help achieve the performance objectives.

### Step 3: Program Design

In Step 3, we conceptualized and designed the program, selecting theory- and evidence-based change methods to achieve the change objectives determined in Step 2. Theoretically, grounded change methods were translated into practical applications, with behavior change techniques (BCTs) [23] systematically applied. This phase focused on ensuring that each component of the program was based on evidence-based methods and tailored to meet the specific needs of youth in out-of-home care. Key tasks included identifying and selecting appropriate change methods based on established theories like the transtheoretical model (TTM), social cognitive theory (SCT), and elaboration likelihood model (ELM).

The TTM was chosen for its effectiveness in promoting behavior change through consciousness-raising and reinforcement management. Liu et al [24] demonstrated that TTM-based interventions help individuals understand the benefits of behavior change and maintain positive habits. By applying TTM, we aimed to support users in recognizing the value of structuring their daily routines and encouraging long-term adherence to healthy behaviors.

The SCT was incorporated for its focus on self-efficacy and observational learning. The theory postulates that internal processes, such as goal setting and self-efficacy, lead to behavioral outcomes [25], which informed our decision to apply SCT. This model supports users in setting goals, building self-efficacy, and learning emotion regulation strategies by observing others.

The ELM was selected to enhance emotional recognition through repeated exposure to emotional stimuli. Petty and Cacioppo [26] emphasized that repeated messaging can significantly influence attitudes and behavior, making ELM a suitable model for improving users’ emotional awareness and regulation. This theory helps explain how consistent exposure to emotional content can lead to deeper emotional understanding and behavior change.

These theoretical methods were then translated into practical applications by designing specific features within the mobile app to deliver the selected change methods effectively. We prioritized a user-centered design approach, as recommended by Johnson et al [27] and Bakker et al [28], for enhancing the app’s usage and effectiveness. The practical applications were developed to be user-friendly and engaging, allowing users to easily interact with the app and incorporate desired behaviors into their daily routines. The aim was to create a structured theoretical framework to help youth in out-of-home care manage their daily lives independently and improve their emotion regulation abilities.

### Step 4: Program Production

Each component of the intervention app was developed systematically by integrating theoretical foundations, designing the user interface, and implementing functionalities. This step aimed to ensure the app’s practical applicability and functionality. Educational content, messages, and images depicting characters and emotions were also developed in a structured manner. An iterative development process was employed, working with the app development team to check and revise the prototype. Regular meetings were held with the lead app developer to communicate requirements, review screen designs, and discuss necessary modifications. These discussions were essential to consider and finalize the design modifications and ensure that the app met all the requirements.

### Step 5: Program Implementation Plan

The fifth step involved creating a comprehensive program implementation plan. This plan included strategies to ensure that the mobile app, which was designed to improve the mental health of youth in out-of-home care, was effectively adopted, used, and maintained over time.

### Step 6: Evaluation Plan

The sixth step was to develop a plan to evaluate if the app had achieved its goals and objectives. This plan focused on conducting a thorough assessment of the app’s effectiveness, covering both process and outcome evaluations.

### Ethical Considerations

This study was approved by the Eulji University Institutional Review Board (approval EU23-43). Participants in the needs assessment interviews received detailed information about the study, and written consent was obtained before their participation. They were assured of their right to withdraw consent verbally at any stage of the interview, even after it had started, without facing any negative consequences. All collected data were anonymized and securely stored in encrypted files, accessible only to the research team. As an expression of gratitude, participants were offered a gift card worth 50,000 Korean won (approximately $35 USD).

## Results

### Step 1: Logical Model of the Problem

Our literature review revealed that young people living in youth shelters often experience a high prevalence of mental health issues, like anxiety, depression, suicidal thoughts, and a poor quality of life [4]. After leaving youth shelters, they often experience isolation, loneliness, despair, and suicidal thoughts as they navigate the challenges of independent living [7].

Individual interviews were conducted with 5 adolescents aged 18-23 years who had been living in shelters for 5 months to 5 years. Many of the participants appeared to be either overweight or obese. Participants have experienced feelings of loneliness, depression, and, in some cases, suicidal thoughts. When facing difficult times, they sought support primarily from their romantic partners, peers, and shelter staff. When asked about their biggest concerns regarding independent living, financial stability was the primary worry, especially related to securing housing and managing living expenses after leaving the shelter. Day-to-day challenges, such as waking up on time and preparing meals, were significant concerns for many due to their limited experience in these areas. They described their current unstructured daily lifestyle—waking up late, spending most of their time lethargically in the shelter, and frequently looking at their smartphones. This reflects a lack of knowledge about healthy daily routines and the absence of motivation to maintain them (lack of knowledge about the advantages of daily routines, lack of motivation to structure daily routines). Psychologically, they expressed fears of dealing with loneliness after becoming independent. They mentioned that they would appreciate having a mobile app they could use daily, especially one that included visual elements like characters to make the experience more engaging.

Focus group interviews were conducted with shelter staff between the ages of 29 and 59 years, who had 1 to 9 years and 11 months of work experience. They specialized in counseling psychology, social welfare, youth education, counseling, and youth studies. The staff explained that the mental health of adolescents entering shelters recently was worse compared to those in the past, with many experiencing higher levels of depression and anxiety. Most adolescents spend the majority of their day on their phones, engaging in social media, calls, YouTube videos, and games. During their days off, they often wake up around 1-2 PM, have meals at the shelter, socialize with friends, and return just before bedtime. This indicates a lack of motivation to adopt healthy behaviors, such as exercise or balanced meals, and the absence of habits that support a healthy lifestyle (lack of knowledge about the importance of healthy behaviors, lack of habit in maintaining healthy behaviors).

The staff observed that some adolescents at the shelter have difficulties with emotional regulation, including depression and anxiety, and actively seek counseling. At times, some adolescents stay up past bedtime due to self-harm urges and turn to teachers for assistance. They also discussed how adolescents struggle to regulate their emotions even in the presence of peers and teachers. Furthermore, when these adolescents transition to living independently, they experience loneliness and depression, prompting them to seek support. This is connected to the lack of strategies for emotional regulation and the absence of motivation to implement those strategies consistently (lack of knowledge about emotional regulation strategies, lack of motivation to regulate emotions).

The staff mentioned that some adolescents struggle to manage their daily lives and emotions even after becoming independent. In some cases, they choose to return to the shelter. In addition, some adolescents do not maintain contact after leaving. Therefore, the staff suggested that a mobile app could assist youth in managing their daily lives and emotions postindependence. The program will help youth develop the knowledge and habits necessary for managing their emotions, as well as motivate them to consistently track and regulate their emotional states (lack of habit in tracking emotions, lack of acceptance of one’s emotions).

**Figure 1:**
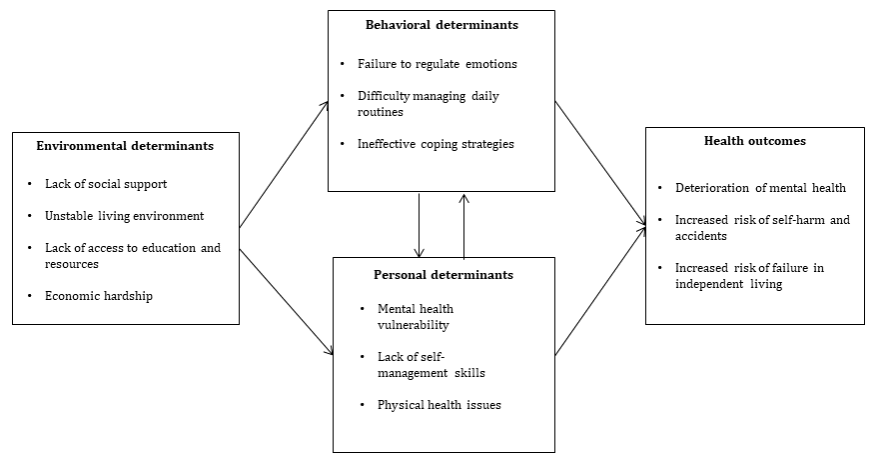
Logical model of the problem.

Out-of-home youth living in shelters encounter challenges due to a lack of support from family and caregivers, resulting in feelings of anxiety, depression, and a tendency to engage in self-injury. In their transition to independent living, many youth face loneliness, leading to feelings of powerlessness, difficulties in daily life management (lack of structured routines, poor habit formation), and struggles with emotional regulation (lack of emotional regulation strategies, lack of motivation). The proposed program aims to empower youth in out-of-home care to address these challenges by effectively managing their daily lives, regulating their emotions, and improving their stability and emotional well-being (Figure 1).

### Step 2: Program Outcomes and Objectives: Logical Model of Change

Based on the findings of the initial phase, the expected program outcomes were: (1) young people managing their daily lives independently and (2) young people enhancing their emotion regulation. Specific performance objectives for independently managing daily lives included structuring and maintaining daily routines, as well as developing healthy habits. In addition, performance objectives for enhancing emotion regulation in young people involved in expressing their emotions and implementing strategies for emotion regulation.

There is evidence that knowledge influences attitude, leading to behavioral changes. Habit formation is crucial for lasting behavioral change, especially when dealing with changing attitudes and temptations [29]. In this study, knowledge, attitude, and habits were chosen as determinants. For out-of-home youth with little family and adult support, forming habits is a key factor in being able to manage daily life and emotions independently. The change objectives are outlined for each of the four performance objectives in the three determinant areas (Table 1).

**Table 1:** Matrix of change objectives.

Outcome and PO^a^		Determinant		
		Knowledge or cognition	Attitude or motivation	Habit
**Young people manage their daily lives independently**				
	PO 1. Young people structure and maintain their daily routines (e.g., time management, scheduling)	K.1. Identify the advantages of structuring daily routines	A.1. Recognize the advantages of structuring daily routines	H.1.a. Monitor the progress of achievement of daily routines H.1.b. Stick to routines to turn them into habits
	PO 2. Young people adapt healthy behaviors (e.g., regular exercise, balanced diet)	K.2. Identify the importance of healthy behaviors	A.2. Recognize the importance of healthy behaviors	H.2.a. Monitor the progress of achievement of healthy behaviors H.2.b. Stick to healthy behaviors to turn them into habits
**Young people enhance their emotion regulation abilities**				
	PO 3. Young people express their emotions	K.3. Identify and name a range of emotions	A.3. Express an accepting attitude towards one’s own emotions	H.3.a. Incorporate emotional expression into daily routines H.3.b. Track one’s own emotions
	PO 4. Young people implement strategies to regulate emotions	K.4. Identify the strategies to regulate one’s own emotions	A.4. Express an attitude of openness to regulate emotions	H.4. Incorporate emotion regulation into daily routines

^a^PO: performance objective.

### Step 3: Program Design

The theory-based program design and practical applications to achieve change objectives are detailed in Table 2. Core components of the program include structuring and maintaining routines, and regulating emotions delivered by a smartphone app. The design was based on behavioral change theories to provide users with tools to manage their daily lives and emotions. BCTs [23] were applied to operationalize the theoretical methods and provide structured guidance to users.

**Table 2:** Explanations of changeable determinants, theory- and evidence-based change methods, and practical applications.

Determinant, change objective, and theory				Method		BCTs^a^		Practical application		Text description
**Knowledge or cognition**										
	K.1. Identify the advantages of structuring daily routines K.2. Identify the importance of healthy behaviors									
		Transtheoretical model	Consciousness raising		4.1 Instruction on how to perform a behavior		Intro session with educational messages on the app		The intro session provides users with educational messages explaining the importance of structuring daily routines and maintaining healthy behaviors.	
	K.3. Identify and name a range of emotions									
		Elaboration likelihood model	Message repetition		4.1 Instruction on how to perform a behavior		Emotion flashcards		Flashcards repeatedly show various emotions to help users recognize and label their emotions more accurately.	
	K.4. Identify strategies to regulate one’s own emotions									
		Social cognitive theory	Observational learning		2.1 Others monitoring with awareness		Observing a character model regulating their emotions		Users learn emotion regulation strategies by observing how character models handle their emotions.	
**Attitude or motivation**										
	A.1. Recognize the advantages of structured daily routines A.2. Recognize the importance of healthy behaviors									
		Social cognitive theory	Goal setting		1.1 Goal setting (behavior)		Setting daily routines, including healthy behaviors		Users set personal goals for structured routines and healthy behaviors, such as regular exercise and a balanced diet, to develop positive habits	
	A.3. Express an accepting attitude towards one’s own emotions									
		Elaboration likelihood model	Message repetition		5.4 Self-assessment of affective consequences		Selecting an emotion flashcard and choosing the corresponding word for the emotion		The flashcards and repeated exposure help users build a positive attitude toward acknowledging their emotions.	
		Social cognitive theory	Observational learning		3.3 Social support (emotional)		Character showing empathy toward their emotions		Character models in the app show empathy towards users’ emotions, helping to build a supportive and motivating environment for emotion regulation.	
	A.4. Express an attitude of openness to regulate emotions									
		Social cognitive theory	Verbal persuasion		15.1 Verbal persuasion to boost self-efficacy		Offering positive reinforcement through verbal encouragement		Verbal encouragement, such as “you can do it,” boosts users’ confidence in their ability to regulate emotions and motivates them to make initial efforts.	
**Habit**										
	H.1.a. Monitor the progress of achievement of daily routines H.1.b. Stick to routines to turn them into habits H.2.a. Monitor the progress of achievement of healthy behaviors H.2.b. Stick to healthy behaviors to turn them into habits									
		Social cognitive theory	Self-monitoring		2.3 Self-monitoring of behavior		Tracking daily achievements using a calendar function		Users track daily routines using a calendar that displays progress from the start of the month to the present day. Progress is visualized with a cake filling piece by piece on the daily screen.	
		Social cognitive theory	Feedback		2.2 Feedback on behavior		Informing and displaying routine accomplishments		The program provides feedback by completing the cake visual when all daily routines are achieved, motivating users to maintain routines.	
		Transtheoretical model	Reinforcement management		10.2 Material reward		Rewards for maintaining routines; points for achievements		Users earn points for maintaining routines and healthy behaviors, reinforcing positive habit formation.	
	H.3.a. Incorporate emotional expression into daily routines H.3.b. Track one’s own emotions									
		Social cognitive theory	Feedback		2.2 Feedback on behavior		Notifications to encourage the expression of emotions		If users do not log their emotions, the app sends a pop-up notification reminding them to record their emotions as part of their daily routine.	
		Social cognitive theory	Self-monitoring		2.4 Self-monitoring of the outcome of behavior		Visualizing weekly emotions in a graph		Users can monitor their emotions through visual graphs that summarize their emotional states over time, encouraging self-reflection and awareness.	
	H.4. Incorporate regulating emotions into daily routines									
		Transtheoretical model	Reflection		15.4 Self-talk		Keeping an emotion diary		Users keep an emotion diary to reflect on their emotional states, helping to improve emotional regulation.	
		Transtheoretical model	Reinforcement management		10.2 Material reward		Points for completing each routine task and diary entry		Users earn points for completing routine tasks and maintaining an emotion diary, encouraging consistency in emotional regulation.	

^a^BCT: behavior change technique.

Consciousness-raising techniques from the TTM helped users understand the benefits of organizing routines and fostering healthy behaviors [30]. Emotion recognition was enhanced using the ELM through repeated exposure to emotion flashcards [26]. Observational learning from the SCT was applied by character models demonstrating emotion regulation [25].

To further enhance motivation, the SCT’s goal setting allowed users to establish routines and healthy behaviors independently, while verbal persuasion and supportive character models built confidence in emotion regulation. Self-monitoring and reinforcement strategies from the SCT and TTM supported habit formation and emotional regulation [31,32].

These theoretical methods were operationalized into practical strategies through the application of BCTs [23], including goal setting, self-monitoring, and reinforcement, which facilitated users in adopting and sustaining behavior changes in their daily routines.

### Step 4: Program Production

**Figure 2:**
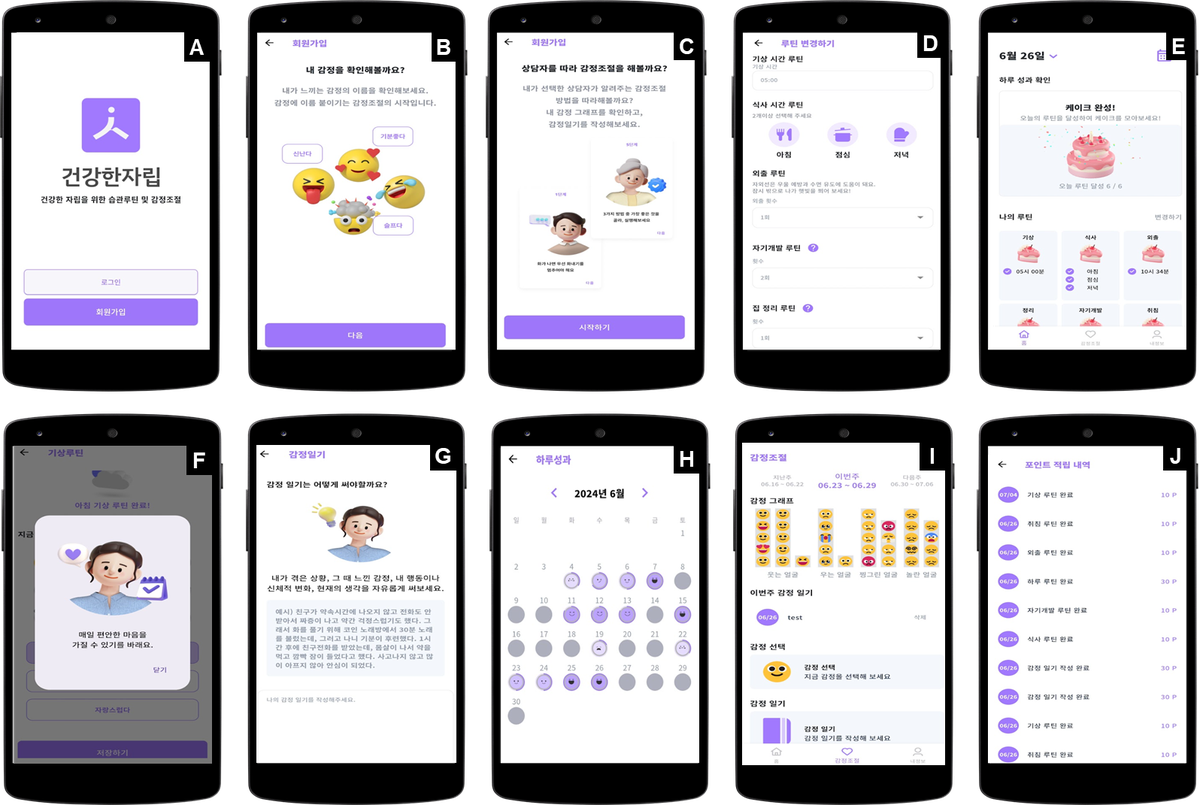
Overview of the app interface. A: Login screen. B, C: Introduction to the app’s purpose and features for managing routines and regulating emotions. D: Screen for setting and modifying daily routines, such as wake-up time, bedtime, meal frequency, and personal development activities. E: Visual representation of daily achievements using a cake image that fills as routines are completed. F: Feedback section where users select their feelings after completing routines and receive messages from a counselor. G: Emotion diary for recording and managing feelings. H: Calendar that shows monthly progress. I: Graph that visualizes emotional changes over time. J: Points section where users can view and track points earned for completing routines.

The fourth step involved developing the specific components of the program to give it a viable form. To create an app for youth in out-of-home care, the program was designed by incorporating information gathered in Steps 1 to 3. Prototypes were utilized in designing the app’s structure and main features, focusing on making the interface intuitive and visually appealing for easy use by the youth. Figure 2 offers an overview of the mobile app’s user interface, showcasing the main components and navigation elements. It presents the home screen comprehensively, including the dashboard, navigation menu, and key interactive features like touchable buttons and icons.

Once users sign in and provide their information, they can use the app after obtaining approval from the administrator. Approved users receive guidance on using the app, including details regarding the importance of daily routines and healthy behaviors. Users then select a character to represent themselves and another character to act as their counselor. Subsequently, they set their daily routines, which include wake-up time, bedtime, number of outings per day, number of meals per day (at least 2), personal development activities (eg, reading, exercising, and studying), and household chores (eg, cleaning and washing dishes). The default home screen displays the current date and provides visual feedback, showing a cake image that is filled in based on routine completion. In addition, the calendar function allows users to visually track their daily achievements and serves as a self-monitoring tool to easily observe their progress.

During the day, users can input whether they completed their routines and the time spent doing so. They can then select their emotions from a list of 12 emoticons, along with appropriate verbal expressions. The counselor’s character provides messages based on the selected emotions to create an emotionally supportive environment. For instance, if a user chooses “anxious,” they receive advice such as, “Something is making you anxious. Consider the causes and prioritize tasks to write down. Talking or writing about your problems can help calm your negative emotions.” This can aid users in recognizing and managing their emotions.

The Emotion Regulation tab shows facial images gathered over a week in a graph, allowing users to visually monitor their emotional changes. In addition, users can write emotion diaries to document and analyze their feelings, aiding in improved understanding and management of emotions.

In the My Info tab, users can view basic information such as their characters, accumulated points, and set routines. They can also check and modify their routines at any time. The Settings tab offers options to change passwords, set notifications, and log out or reset accounts.

The development of the mobile intervention app followed a structured process that included defining requirements, screen planning, development, testing, and deployment. The development environment consisted of both a web-based system for data and user management and a mobile application. The mobile application was developed as a hybrid app designed for both Android and iOS platforms to maximize accessibility for users. The app was registered and made available through the Google Play Store and Apple App Store. User data was securely stored in the MySQL database, and the server was hosted on Amazon Web Services to ensure robust data protection. Security measures were carefully implemented to protect user information. User authentication was managed using JSON Web Token, providing a secure method for validating user sessions. All data transmissions were encrypted using SSL/TLS through the HTTPS protocol, ensuring that sensitive information remained protected during communication between the app and the server. In addition, server access control was strictly managed to further enhance security.

### Step 5: Program Implementation Plan

The fifth step is a detailed plan on how we envision the adoption, implementation, and long-term maintenance of the app within shelter settings. The program uses outcomes (adoption and implementation) that were developed with specific performance objectives for both the shelter staff and youth. Using normalization process theory [33], we identified changeable determinants that could influence the successful integration of the app into daily routines. These determinants were crossed with performance objectives to establish clear change objectives. The theoretical foundation for our strategies is based on BCTs [23], which informed the methods for fostering app adoption and ensuring its sustained use. Practical strategies include training sessions for staff, peer ambassador programs, and motivational tools for the youth, which were all designed to encourage continuous engagement with the app. Table 3 outlines the detailed performance objectives, change objectives, and corresponding strategies for both adoption and implementation.

**Table 3:** Plan for program adoption and implementation.

Program use outcomes, performance objectives, and determinants (NPT^a^)				Change objectives		BCTs^b^	Practical strategies
**Adoption outcome**							
	**Shelter staff adopt the app**						
		Coherence: Staff understand the benefits of the app	Staff will develop an understanding of how the app supports mental health management		4.1 Instruction on how to perform a behavior		Provide informational sessions and materials on the app’s benefits for mental health.
	**Staff encourage youth to use the app regularly**						
		Cognitive participation: Staff engage in the program	Staff will actively promote app use to youth in their daily routines		1.8 Behavioral contract		Staff will commit to a plan to introduce the app to youth and track usage
		Collective action: Staff train youth on app use	Staff will train youth to use the app and integrate it into daily life		4.1 Instruction on how to perform a behavior		Staff will provide step-by-step training on app functionality and its benefits.
**Implementation outcome**							
	**Youth use the app daily**						
		Coherence: Youth understand how the app helps manage emotions	Youth will understand the purpose of the app and its features		4.1 Instruction on how to perform a behavior		Educational sessions and motivational posters will explain the app's purpose.
		Cognitive participation: Youth engage in regular use	Youth will commit to using the app daily to track emotions and routines		8.1 Behavioral practice or rehearsal		Youth will be encouraged to practice app use through daily emotional logging and tracking routines.
		Reflexive monitoring: Youth track progress and give feedback	Youth will evaluate how the app helps them manage their daily routines		2.3 Self-monitoring of behavior		Youth will log daily routines and emotions; feedback will be collected for improvement.
**Sustainability**							
	**Maintain app usage long-term**						
		Collective action: Peer ambassadors promote the app	Ambassadors will share success stories to encourage long-term app use		6.3 Information about others’ approval		Peer ambassadors will share their experiences and offer support through group sessions.
		Reflexive monitoring: Continuous improvement	Youth will provide feedback for app improvements		1.5 Review of behavior goals		Regular feedback sessions will be held to improve app functionality and user experience.

^a^NPT: normalization process theory.

^b^BCT: behavior change technique.

### Step 6: Evaluation Plan

The sixth step involved creating a detailed and comprehensive plan to evaluate the designed mobile app. This evaluation plan aims to systematically analyze the implementation process and effectiveness and identify areas for improvement, if necessary.

#### Process Evaluation

Process evaluation confirms whether the app is being implemented as planned and assesses the fidelity of its implementation. Key indicators include the number of youths using the app, frequency of app usage, levels of user engagement, and the frequency with which users use the key features of the app. These data will be collected through various methods, including app usage analytics software, user logs, and feedback surveys from youth residing in shelters. Real-time data collection will be implemented to analyze usage patterns and engagement levels. User logs will track individual user activities to help identify the most and least used features. Regular feedback surveys will assess subjective user experiences and satisfaction, covering aspects such as ease of use, usefulness of features, and overall satisfaction. In addition, in-depth interviews will be conducted to gain a more detailed understanding of user experiences, focusing on the strengths, weaknesses, and areas for improvement of the app. This comprehensive data collection will help determine if the app is being implemented as planned, identify any unforeseen issues, and understand the most beneficial features to users.

#### Effectiveness Evaluation

The program’s effectiveness will be assessed through a randomized controlled trial. Participants will be assigned randomly to either the experimental or control group using computer-generated random allocation. The experimental group will receive the app intervention, while the control group will receive standard care provided by the shelters during the trial. In consideration of ethical concerns, the control group will be given access to the app after the intervention period has ended, ensuring that all participants can benefit from the app. The evaluation will focus on the app’s effects on youth’s mental health, daily living skills, and emotion regulation. Key measures will include decreases in anxiety and depressive symptoms, better emotion regulation, and improved daily living skills. Data collection methods will include pre- and post-surveys, standardized mental health assessment tools (such as the Beck Depression Inventory and Generalized Anxiety Disorder Scale), and user interviews.

Quantitative data analysis will be conducted using IBM SPSS (version 26). The 1-way, repeated-measures, multivariate ANOVA will be used to assess the intervention effects. Qualitative data from interviews and open-ended survey responses will undergo thematic analysis to gain deep insights into user experience and app effectiveness. This thorough evaluation plan aims to systematically evaluate the app’s implementation process and outcomes, aiming to enhance youth’s mental health. Our goal is to optimize the app’s effectiveness and promptly make any necessary improvements.

## Discussion

### Principal Results and Comparisons With Previous Work

A mobile app was developed in this study to enhance the mental health of youth in out-of-home care using the IM approach. The app was created through a systematic and step-by-step development process.

Earlier studies have used the IM approach to create successful health promotion programs for different health concerns [19-21]. Nonetheless, IM-based mental health promotion initiatives aimed at youth residing in shelters are scarce. Furthermore, programs developed in previous studies frequently depend on brief interventions centered on in-person counseling, posing challenges in maintaining lasting impacts [12,17]. In South Korea, mental health apps such as MindLink [34] and Mind Café provide early intervention and emotional support to youth, but they are not specifically designed for youth residing in shelters. MindLink, for example, focuses on psychosis intervention, while Mind Café provides short-term emotional support through counseling, but it may be limited due to cost-related barriers to sustained access. These apps are valuable for their specific purposes but lack tailored features to address the unique needs of youth in out-of-home care. The mobile app developed in this study was created to overcome these limitations by providing accessible emotional support and daily management functions to youth. Emotion flashcards and self-monitoring tools assist users in recognizing and regulating their emotions independently, helping them cultivate their ability to self-manage even after leaving the shelter.

Our program was designed after conducting a comprehensive needs assessment of youth living in shelters. A previous study has emphasized the importance of a thorough needs assessment to improve program effectiveness [35]. This study investigated the behavioral and environmental factors influencing mental health issues by reviewing literature and interviewing youth in out-of-home care and shelter staff with the aim of providing tailored interventions.

We designed a program using theories like the TTM, SCT, and ELM to improve the scientific validity of the program’s mental health promotion program. By integrating these theories, our program identified knowledge, attitudes, and habit formation as key determinants, applying each theory’s methods to enhance the app’s effectiveness. Previous studies that incorporated these theories have shown positive outcomes.

We prioritized a user-centered design approach to enhance the app’s usage and effectiveness. To increase youth’s engagement and persistence, we implemented various strategies. For instance, users have the option to personalize their experience by selecting both their own character and their counselor’s character. The app’s user-friendly design, combined with a visual reward system for completing daily tasks, sparks interest and boosts engagement, encouraging continuous use. Users can establish their daily routines and receive visual cues indicating progress, such as a cake image that fills up as they complete tasks, providing a sense of achievement. When users choose an emotion from a list, the counselor responds with appropriate messages to foster emotional support. For example, if a user selects “anxious,” the counselor’s character might suggest, “Identify what’s causing your anxiety and focus on tackling simple tasks first. Acknowledging and addressing your problems can help alleviate negative feelings.” This feature enhances emotional support and equips users with tools to manage their emotions effectively. The Emotion Regulation section displays facial expressions accumulated over a week, allowing users to monitor changes in their emotions. By keeping emotion diaries, users can record and reflect on their feelings, which enhances their emotional awareness and regulation skills.

### Limitations

The interviews conducted during the needs assessment of this study’s development process focused on gathering real-world needs and collaborating with stakeholders. Therefore, insights obtained from these interviews were integrated iteratively, and any emergent trends were reviewed with the planning group. However, using formal analysis frameworks in future projects could enhance rigor and reliability. This study focused on developing a mobile app but did not confirm its effectiveness through application and evaluation. This limitation hinders the assessment of the app’s effectiveness and user satisfaction. Future research should validate the app’s long-term effectiveness in real-world scenarios. The study specifically targeted youth living in shelters in a specific region, limiting the generalizability of the findings to youth in other regions and cultural contexts. Further research is necessary to evaluate the app’s use among youth from various regions and cultural backgrounds.

### Conclusions

This study outlines the development process of a mobile app aimed at enhancing the mental health of youth in out-of-home care using the IM approach. The app was created to assist youth in managing their daily lives and regulating their emotions independently, offering high accessibility and continuous support. The systematic development process and theoretical foundations for designing the app’s structure and features are described, along with plans for evaluating its process and effectiveness.

Although real-world use of the app was not evaluated in this study, the systematic approach and substantial stakeholder involvement in the development phase indicate its potential effectiveness. Future research should focus on assessing the app’s real-world usage and incorporating user feedback for continuous improvement. Additional studies are anticipated to establish a strong foundation for developing and implementing digital health interventions that can significantly improve the mental health and well-being of youth in out-of-home care.

## Abbreviations

BCT: behavior change technique
ELM: elaboration likelihood model
IM: intervention mapping
SCT: social cognitive theory
TTM: transtheoretical model

## Data Availability

The data that support the findings of this study are available from the corresponding author upon reasonable request.
